# Impact of genetic variants within serotonin turnover enzymes on human cerebral monoamine oxidase A in vivo

**DOI:** 10.1038/s41398-023-02506-2

**Published:** 2023-06-15

**Authors:** Marie Spies, Matej Murgaš, Chrysoula Vraka, Cecile Philippe, Gregor Gryglewski, Lukas Nics, Theresa Balber, Pia Baldinger-Melich, Annette M. Hartmann, Dan Rujescu, Marcus Hacker, Edda Winkler-Pjrek, Dietmar Winkler, Rupert Lanzenberger

**Affiliations:** 1 Department of Psychiatry and Psychotherapy, Comprehensive Center for Clinical Neurosciences and Mental Health (C3NMH), Medical University of Vienna, Vienna, Austria; 2 Department of Biomedical Imaging and Image-guided Therapy, Division of Nuclear Medicine, Medical University of Vienna, Vienna, Austria; 3 Child Study Center, Yale University, New Haven, CT USA; 4 Ludwig Boltzmann Institute Applied Diagnostics, Vienna, Austria

**Keywords:** Molecular neuroscience, Medical genetics, Depression

## Abstract

Variants within the monoamine oxidase A (MAO-A, *MAOA*) and tryptophan hydroxylase 2 *(TPH2*) genes, the main enzymes in cerebral serotonin (5-HT) turnover, affect risk for depression. Depressed cohorts show increased cerebral MAO-A in positron emission tomography (PET) studies. *TPH2* polymorphisms might also influence brain MAO-A because availability of substrates (i.e. monoamine concentrations) were shown to affect MAO-A levels. We assessed the effect of *MAOA* (rs1137070, rs2064070, rs6323) and *TPH2* (rs1386494, rs4570625) variants associated with risk for depression and related clinical phenomena on global MAO-A distribution volume (V_T_) using [^11^C]harmine PET in 51 participants (21 individuals with seasonal affective disorder (SAD) and 30 healthy individuals (HI)). Statistical analyses comprised general linear models with global MAO-A V_T_ as dependent variable, genotype as independent variable and age, sex, group (individuals with SAD, HI) and season as covariates. rs1386494 genotype significantly affected global MAO-A V_T_ after correction for age, group and sex (*p* < 0.05, corr.), with CC homozygotes showing 26% higher MAO-A levels. The role of rs1386494 on TPH2 function or expression is poorly understood. Our results suggest rs1386494 might have an effect on either, assuming that TPH2 and MAO-A levels are linked by their common product/substrate, 5-HT. Alternatively, rs1386494 might influence MAO-A levels via another mechanism, such as co-inheritance of other genetic variants. Our results provide insight into how genetic variants within serotonin turnover translate to the cerebral serotonin system. Clinicaltrials.gov Identifier: NCT02582398. EUDAMED Number: CIV-AT-13-01-009583.

## Introduction

Altered serotonin (5-HT) turnover, the relationship between synthesis and degradation, is implicated as a central pathophysiologic mechanism in depression. Serotonin degradation is amplified in individuals with depression, particularly in the presence of increased genetic risk, as demonstrated by brain efflux of 5-HT metabolites [1]. Accordingly, 5-HT synthesis was shown to be decreased in depression [2] and increased after antidepressant treatment [3], the latter of which may result from up-regulation of tryptophan hydroxylase (TPH) expression [4]. TPH is the rate-limiting enzyme in serotonin synthesis [5]. Its second isoform, TPH2, is essential to 5-HT production in the brain [6], but also regulates noradrenaline (NE) and dopamine (DA) levels [5]. 5-HT degradation, but also that of NE and DA, is fundamentally regulated by the monoamine oxidase - A (MAO-A) [7].

Positron emission tomography (PET) allows for highly specific human in vivo assessment of 5-HT turnover and provides evidence for altered 5-HT metabolism with decreased synthesis and increased degradation in depression. Decreased serotonin synthesis rates were shown using α-[^11^C]methyl-L-tryptophan PET [2]. Increased brain MAO-A levels were demonstrated using [^11^C]harmine PET [8–10]. PET studies also suggest that the enzymatic processes regulating 5-HT synthesis and degradation are linked, with brain MAO-A V_T_ shown to be related to levels of its substrates, including 5-HT [11].

Genetic variants within the genes coding for the central monoamine-turnover proteins MAO-A (*MAOA*) and TPH2 (*TPH2*) have been linked to risk for depressive disorders and related phenomena. The *MAOA* single nucleotide polymorphisms (SNPs) rs1137070 (T/C, exonic, in high linkage disequilibrium with rs2064070 (A/T, downstream variant)) and rs6323 (G/T, exonic) have been associated with depression [12, 13] and suicidal symptoms [14], respectively. Similarly, the common *TPH2* SNPs rs1386494 (T/C, intronic) [15] and rs4570625 (T/G, promotor) [16, 17] are among those within this gene most decisively linked to depression. Their respective C (rs1386494) [15] and G (rs4570625) [16] alleles were associated with disease risk, while rs1386494 T homozygosity was linked to improved antidepressant treatment response [18]. In addition, rs4570625 was repeatedly shown to mediate amygdala reactivity [19–21], changes to which are understood as a fundamental finding in depression [22]. However, the molecular functional relevance of these variants remains unclear. In vitro studies associate higher mRNA levels and higher MAO-A activity with the *MAOA* rs1137070 T [23] and rs6323 G [24] alleles, respectively. However, the effect of these variants on human in vivo protein levels and the extent to which MAO-A V_T_ may be a relevant endophenotype for their clinical effects, have yet to be assessed.

Light and season also affect 5-HT turnover [25]. Seasonal and meteorologic changes are implicated in seasonal affective disorder (SAD), a subtype of depression characterized by depressive symptoms in fall/winter and remission in spring/summer, for which underlying serotonergic pathophysiology is presumed [26]. However, the relevance of the serotonin turnover genetic variants addressed here have not be studied in SAD. Furthermore, while we previously reported unaltered MAO-A V_T_ in SAD, the extent to which genetic factors impacting on MAO-A V_T_ might increase variability, hereby obscuring MAO-A V_T_ group differences, is unclear.

Here we test the effect of *MAOA* and *TPH2* SNPs on MAO-A levels assessed using [^11^C]harmine PET, based on the tight interplay between enzymatic processes regulating serotonin homeostasis. We test effects on MAO-A levels in individuals with SAD and healthy individuals (HI) based on reports linking these variants to depression [12, 13, 15–17], insufficient information on their relevance to SAD, as well as sensitivity of 5-HT turnover to season and light [25].

## Materials and methods

### Study design

Here we assessed 21 individuals with SAD (6/15 male/female, mean age ± SD = 33.43 ± 10.24) and 30 HI (13/17, 33.80 ± 9.76). All individuals with SAD and 23 HI were previously assessed in a study on changes to MAO-A V_T_ measured with [^11^C]harmine PET [27]. The previous study was comprised of three PET measurements, two in fall/winter (once before, and once after bright-light treatment) and one in spring/summer [27]. All subjects from the original study in whom genetic data was available were included in the study at hand. For this analysis, based on data availability, we utilized baseline [^11^C]harmine PET data acquired in fall/winter for all individuals with SAD and 26 HI. For the additional 4 HI in whom fall/winter PET data was not available, PET data measured during the spring/summer seasons was used. All study subjects were assessed for eligibility in a screening assessment. In addition, all subjects underwent one MRI for structural co-registration of PET data at any time during study participation. Blood draw for genotyping was performed at any of the study visits. The study was concluded with a follow-up examination. All subjects provided written-informed consent for study participation, the study was approved by the Ethics Committee at the Medical University of Vienna and was performed according to the Declaration of Helsinki.

### Subjects

In all, 21 individuals with unipolar, winter-type SAD and 30 HI were included in this analysis. During the screening assessment, the structured clinical interview for DSM-IV for Axis I Disorders (SCID-1) was performed to diagnose SAD, exclude the presence of psychiatric comorbidities in individuals with SAD, and exclude psychiatric disorders in HI. Absence of severe internal or neurologic disorders was confirmed by medical history, physical assessment, routine blood draw and electrocardiography. Pregnant and breast-feeding women were excluded from participation. Pregnancy was tested for using a urine-pregnancy test. Previous studies have demonstrated effects of smoking [28] and nicotine withdrawal [29] on brain MAO-A. Smoking and substance abuse were considered exclusion criteria and were tested for using urine-cotinine and urine-drug screens, respectively. Individuals with SAD had to have been free from psychopharmacologic medication within the 6 months prior to inclusion.

### PET measurement

[^11^C]harmine PET measurement was performed as described previously [27, 30] at the PET Center, Department of Biomedical Imaging and Image-guided Therapy at the Medical University of Vienna. A GE Advance full-ring scanner (GE Healthcare, Chicago, Illinois) was used. In short, a cannula was placed in a radial artery prior to PET in order to allow for arterial blood sampling, which is necessary for PET data quantification, which was performed as described in Ginovart et al. [31]. [^11^C]harmine (7- [^11^C]methoxy-1-methyl-9*H*-[3,4-*b*]indole) was synthesized according to procedures established at the PET center, Division of Nuclear Medicine, at the Medical University of Vienna and described by Philippe et al. [32]. Following a 5 min transmission scan utilizing Ge-68 rod sources, an intravenous bolus of 4.6MBq/kg body weight [^11^C]harmine was administered. 3D dynamic PET measurement (90 min consisting of 51 successive time frames: 5 s (12 frames), 10 s (6 frames), 20 s (3 frames), 30 s (6 frames), 1 min (9 frames), and 5 min (15 frames)) commenced simultaneously with radioligand administration. Fourier rebinning iterative filtered backprojection algorithm (FORE-ITER) was used for reconstruction to 35 transaxial section volumes (128 × 128 matrix) resulting in a spatial resolution of 4.36 mm full-width at half maximum next to the center of the field of view. Arterial blood was drawn continuously for the first 10 min using an automated blood sampling system, in addition blood samples were drawn manually at standardized intervals (1, 5, 10, 20, 30, 45, 60, 80 min).

### MRI measurement

One T1-weighted structural MRI measurement, which is necessary for co-registration of PET data, was performed in each subject. MRI was performed using either a 3 Tesla PRISMA MR Scanner (Siemens Medical, Erlangen, Germany, 1 × 1 mm voxel size, 1.1 mm slice thickness, 200 slices) or a 3 Tesla Achieva MR Scanner (Philipps, Best, Netherlands, 0.47 × 0.47 mm voxel size, 0.88 mm slice thickness, 180 slices) as described previously [27].

### PET data analysis

First, each PET scan was normalized to Montreal Neurological Institute (MNI) space using SPM12 (Wellcome Trust Centre for Neuroimaging, London, United Kingdom; http://www.fil.ion.ucl.ac.uk/spm/). Each PET was corrected for head motion and co-registered to the T1 structural image. The resulting transformation matrix was then used to normalize the co-registered PET scan to MNI space. Each MR scan was normalized to MNI space utilizing a tissue probability map included in SPM.

Arterial blood samples drawn manually during scanning were processed according to Ginovart et al. [31]. The automated arterial blood sampling system was cross calibrated with the PET scanner and a Wizard 2480 gamma counter (Perkin-Elmer, Waltham, U.S.A.). PMOD 3.509 (PMOD Technologies Ltd., Zurich, Switzerland; www.pmod.com) was used for the subsequent steps. The arterial input function (AIF), which represents non-metabolized [^11^C]harmine in arterial blood as a function of time, was calculated as the product of whole blood activity (fit with 3 exponentials), ratio of plasma to whole blood (linear fit), and the fraction of non-metabolized tracer concentration in arterial plasma (fit with Watabe function).

Voxel-wise quantification of MAO-A V_T_ was performed using Logan plot [33]. The estimated AIF and the time activity curve of thalamus, representing the high uptake region, were used. Regional V_T_ was extracted from the frontal and temporal poles, anterior and posterior cingulate gyri, thalamus, caudate, putamen, hippocampus, and midbrain, all adopted from the Harvard-Oxford cortical structural atlas (http://fsl.fmrib.ox.ac.uk/fsl/fslwiki/Atlases), as well as striatum, which was delineated using an in-house atlas. Then, a global region of interest (ROI), consisting of average of regional V_T_ weighted for ROI size was calculated, based on the high correlation in MAO-A V_T_ between regions (mean spearman correlation coefficients ± SD = 0.95 ± 0.03, range = 0.87−0.98).

### SNP selection

We investigated the influence of genetic variants within genes essentially related to serotonin turnover. TPH2 is the rate-limiting enzyme in serotonin synthesis [5] and MAO-A is the main serotonergic degradation enzyme [7]. For *MAOA*, rs1137070/rs2064070 (in high LD) and rs6323 were assessed. For *TPH2*, rs1386494 and rs4570625 were included.

SNPs were selected based on qualitative literature search (NCBI, https://www.ncbi.nlm.nih.gov, dbSNP, https://www.ncbi.nlm.nih.gov/snp/). Genetic variants were chosen based on existing evidence linking to depression (rs1137070 (*MAOA*), rs1386494 (*TPH2*)) [12, 13, 15], related phenomena like suicidality (rs6323 (*MAOA*), rs1386494 (*TPH2*)) [14, 34, 35], emotion processing (rs6323 (*MAOA*), rs4570625 (*TPH2*)) [19, 20, 35], and/or treatment response (rs6323 (*MAOA*), rs1386494 (*TPH2*)) [18, 36]. Replicated results and results from meta-analyses were prioritized. In addition, the minor allele frequencies (MAFs), and therefore feasibility of investigation in a study of this size, were taken into consideration. Based on the MAF and sample size, subjects were grouped into 2 groups for each genotype. See Table 1 for grouping and sample sizes.

**Table 1:** Genotype grouping and frequencies.

Variant		
*MAOA*	n (m, f)	n (m, f)
rs1137070 (T/C)^a^	CC	CT, TT
	34 (17, 17)	17 (3, 14)
rs6323 (G/T)	TT	TG, GG
	35 (17, 18)	16 (3, 13)
*TPH2*	n	n
rs1386494 (T/C)	CC	CT
	35	16
rs4570625 (T/G)	GG	GT, TT
	28	23

^a^rs1137070 and rs2064070 were in perfect LD, thus only rs1137070 is reported.

*MAOA* SNPs are split by sex based on X-linkage.

### Genotyping

Genotyping of *TPH2* SNPs was performed using the iPLEX assay on the MassARRAY MALDI-TOF mass spectrometer as previously described by Oeth et al. [37]. Allele specific extension products were identified, and genotypes allocated by Typer 3.4 Software (Sequenom, San Diego, CA, USA). All applied quality criteria were met (individual call rate >80%, SNP call rate >99%, identity of genotyped of CEU Trios (Coriell Institute for Medical research, Camden, NJ) with 1000 Genomes database >99%). Here, rs1137070 and rs2064070 were in perfect LD, therefore statistical analyses were performed for rs1137070 only.

### Statistics

Hardy-Weinberg equilibrium (HWE) was tested for using Chi2 testing and Matlab. For *MAOA* variants, testing was performed within sex groups based on X-linkage. All other statistical analyses utilized SPSS Statistics Version 25 (https://www.ibm.com/analytics/at/de/technology/spss/). After confirmation of prerequisites for parametric analysis, effects of SNPs on MAO-A V_T_ were tested for using general linear models (GLM). Prerequisites for GLM were confirmed visually. Individual models comprised global MAO-A V_T_ as dependent variable and genotype as independent variable. The global ROI was defined as outcome parameter based on the high correlation in MAO-A V_T_ between regions. In addition, group (individuals with SAD/HI), age and sex (m/f) were included as covariates based on previous evidence for effects of these factors on MAO-A V_T_ [38]. Multiple testing was corrected for using Holm-Bonferroni. Furthermore, analysis was repeated in an exploratory manner (1) including season as a covariate, based on effects of season on the serotonin system [39] and including (2) genotype × group, and (3) genotype x sex interaction effects to detect potential group or sex specific genotype effects on MAO-A V_T_.

Our primary analysis utilized fall/winter or spring/summer (when fall/winter was not available, n = 4) scans in HI and only fall/winter PET measurements in individuals with SAD. As individuals with SAD are ill in fall/winter and remit in spring/summer, using only fall/winter scans in this group is essential to probe the relevance of the assessed SNPs to SAD pathophysiology. In contrast, HI are healthy across the year, motivating our decision not to discriminate scan-season in this group. However, based on previous reports of seasonal effects within the serotonin system, even in healthy individuals [40], we repeated statistical analyses without the four HI in whom only spring/summer scans were available, i.e., including only fall/winter scans across both groups.

## Results

Frequencies of the assessed variants were in HWE with the exceptions of rs1137070 and rs6323 in males. See Table 1 for genotype grouping and frequencies.

**Fig. 1:**
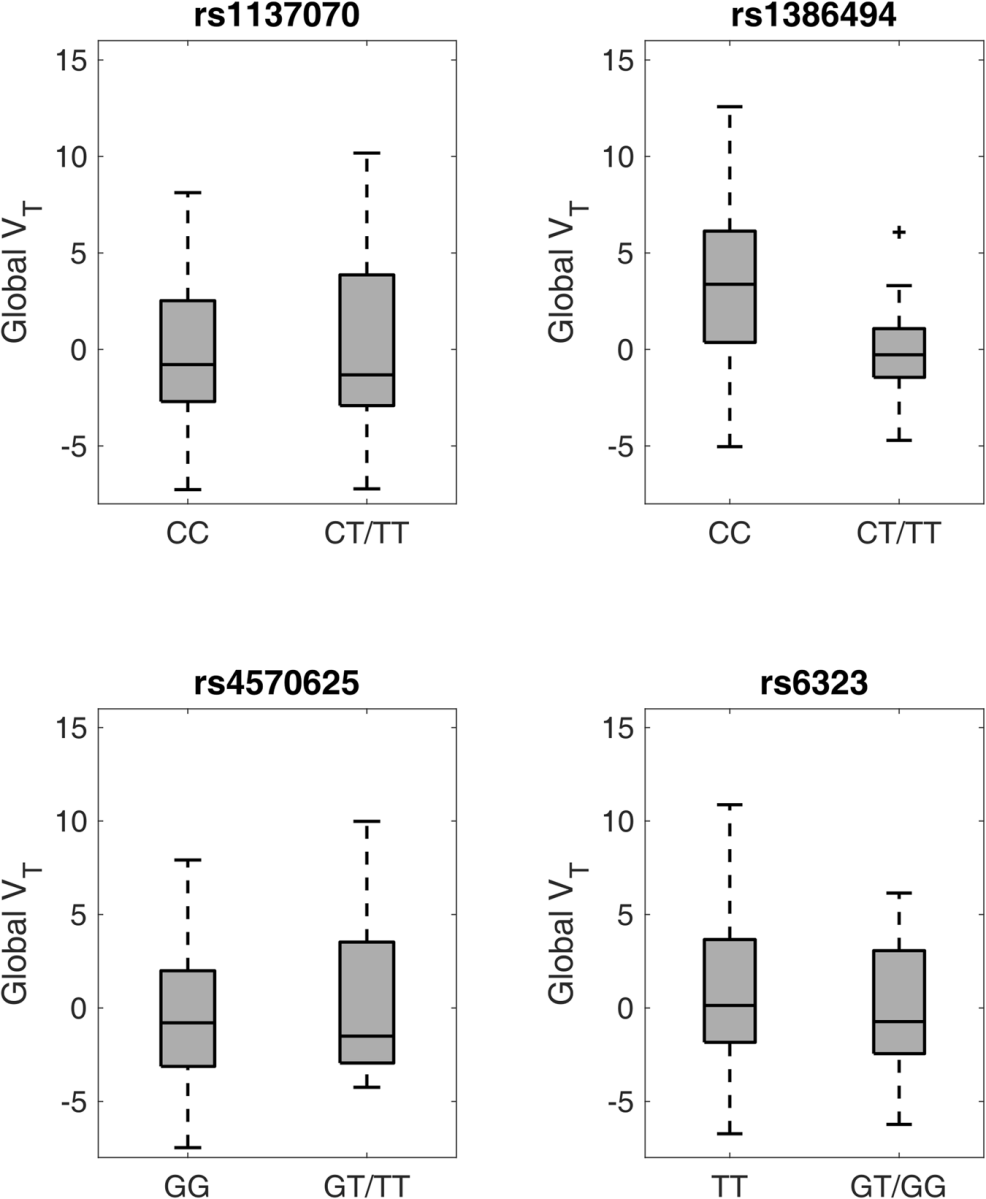
Effects of serotonin turnover variants on MAO-A V_T_. MAO-A V_T_ was significantly (*F*_1, 119.70_ = 9.52, *p*_corr_ = 0.01, cohen’s *d*: 0.88, ~26% numerically) higher in rs1386494 (*TPH2*) CC homozygotes than in T carriers. The other assessed genotypes (*MAOA*: rs1137070, rs6323, and *TPH2*: rs4570625) did not demonstrate statistically significant effects on MAO-A V_T_. MAO-A V_T_ represented here is corrected for age, sex, and group (individuals with SAD/HI). Here, rs1137070 and rs2064070 were in perfect LD, thus only rs1137070 is reported. Boxes represent 50% of data, median depicted, whiskers represent range, + denotes outlier.

rs1386494 (*TPH2*) showed a statistically significant effect on global MAO-A V_T_ (*F*_1, 119.76_ = 9.52, *p*_corr_ = 0.01) with ~26% higher MAO-A V_T_ in CC homozygotes (cohen’s d: 0.88). None of the other assessed genotypes had statistically significant effects on MAO-A V_T_ (cohen’s d: rs1137070: 0.06; rs4570625: 0.11, rs6323: 0.21). See Fig. 1 and Table 2. Neither inclusion of season nor genotype × group or genotype × sex interaction effects as covariates affected results. Interaction effects were non-significant. In addition, age, sex, group, and season were not statistically significant yet retained in the model based on previous evidence [8, 9, 39]. See Table S1 for additional statistical information on GLM models including non-significant results and covariate effects.

**Table 2:** Genotype effects on global MAO-A V_T_.

Variant	Genotype	Global MAO-A V_T_ (mean ± SD)
*MAOA*		
rs1137070 (T/C)^a^	CC	15.01 ± 3.65
	CT, TT	15.24 ± 4.27
rs6323 (G/T)	TT	15.34 ± 4.10
	TG, GG	14.53 ± 3.22
*TPH2*		
rs1386494 (T/C)	CC	16.14 ± 3.85
	CT	12.78 ± 2.65
rs4570625 (T/G)	GG	14.90 ± 3.91
	GT, TT	15.32 ± 3.80

^a^rs1137070 and rs2064070 were in perfect LD, thus only rs1137070 is reported. Values uncorrected for covariates.

Significance levels were unaltered when only including fall/winter PET data (i.e., excluding four HI in whom only spring/summer scans were available). rs1386494 (*TPH2*) again showed a statistically significant effect on global MAO-A V_T_ (*F*_1, 124.31_ = 9.49, *p*_corr_ = 0.02), with CC homozygotes showing 28% higher MAO-A V_T_ (cohen’s *d*: 0.90). Again, none of the other assessed genotypes had statistically significant effects on MAO-A V_T_, interaction effects were non-significant, and their inclusion did not affect results. Also, age, sex, and group did not statistically significantly affect MAO-A V_T_. See Tables S2–S4, and Fig. S1 for additional results pertaining to secondary analysis.

## Discussion

This study demonstrates that a common *TPH2* genetic variant (rs1386494) significantly impacts on human in vivo global MAO-A V_T_ assessed using [^11^C]harmine PET. rs1386494 CC homozygotes showed higher global brain MAO-A V_T_ with a large effect size of cohen’s d: 0.88. These effects were independent of age, sex, season, and group (individuals with SAD, HI). The effect of rs1386494 on cerebral MAO-A expression was thus not impacted by any underlying depressive pathophysiology. The other variants we assessed (*MAOA*: rs1137070/2064070, rs6323, *TPH2*: rs4570625) did not affect brain MAO-A V_T_.

These results are, to our knowledge, the first report of a genetic variant within *TPH2* impacting on human in vivo cerebral MAO-A levels. MAO-A and TPH2 are essential for cerebral serotonin degradation [7] and synthesis [5] and are therefore central to 5-HT turnover. We selected SNPs shown to exhibit clinical effects in affective disorders and related phenomena. The impact of rs1386494 on risk for depression [15] and suicidality [34, 41] as well as on antidepressant response [18] has been demonstrated. However, little is known about the impact of rs1386494 on TPH2 expression and thus effects on brain 5-HT homeostasis.

Our study does not allow for assessment of rs1386494’s effects on TPH2 levels. However, investigation of the effects of 5-HT turnover SNPs on cerebral MAO-A V_T_ was motivated by studies demonstrating that MAO-A and TPH2 activity are, at least indirectly, interrelated. MAO-A V_T_’s dependency on the levels of its substrates may serve as a potential functional link between rs1386494 and MAO-A V_T_. In vivo, tryptophan depletion and carbidopa-levodopa administration were shown to down- and up-regulate MAO-A V_T_ in the PFC and striatum, respectively [11]. On a theoretical level, this relation would suggest that the rs1386494 T allele would be associated with lower TPH2 activity, lower 5-HT levels [5], and subsequently lower MAO-A. In contrast, increased TPH2 activity in the CC group and reversal of the above-mentioned theoretical association also poses a potential mechanism. MAO-A also degrades other monoamines [7] and TPH2 knock-out mice were shown to exhibit deficits in DA and NA synthesis [5]. Therefore, these other monoamines could also play an intermediary role in the link between *TPH2* genotype and MAO-A V_T_.

A theoretical substrate-dependent effect on [^11^C]harmine signal might be dependent on (1) actual changes to MAO-A expression or (2) changes in [^11^C]harmine affinity to the MAO-A, particularly as [^11^C]harmine is a competitive, reversible MAO-A inhibitor [42]. In vitro evidence speaks towards a substrate-dependent effect unrelated to radioligand affinity. More specifically, a D2-receptor-agonist mediated increase in MAO-A activity that was related to inhibition of cAMP and transcriptional changes was shown in rat mesangial cells. These results suggest a positive, [^11^C]harmine independent, substrate-mediated effect, at least for dopamine [43].

On the other hand, the MAO-A V_T_ differences we detected between rs1386494 genotype groups might be mediated by a factor related to rs1386494 but independent of TPH2 or 5-HT levels. Such a factor might be a clinical variable that we were not able to address despite our correction for age, sex, season, medication, and smoking. Furthermore, another genetic variant co-inherited with rs1386494 might in fact facilitate the detected MAO-A V_T_ effects. For example, the *TPH2* variant rs1386493, of which the A allele (correlated with rs1386494 T allele with an *r*^2^ of = 0.814, based on https://ldlink.nci.nih.gov in European populations) results in alternative splicing of TPH2 and reduced enzymatic function [23]. Therefore, though we did not genotype this SNP in our sample and this interpretation is hypothetical, co-inheritance of rs1386493 or other variants with known effects on monoamine protein levels or their function could potentially mediate the rs1386494 related effects we detected.

rs1386494 CC genotype was associated with increased MAO-A V_T_ both in HI and individuals with SAD, and not impacted by inclusion of group as a covariate, even after exclusion of 4 HI in whom only spring/summer scans were available. This suggests that the variant has a general effect independent of underlying depressive pathology. The C allele was previously linked to depression [15], persons with depression show increased MAO-A levels [8, 9], and we found higher MAO-A V_T_ in CC homozygotes. If our results had been restricted to individuals with SAD, one might consider that increased MAO-A V_T_ may function as an endophenotype for rs1386494 mediated clinical effects. Again, our effects were not group-specific, and group (individuals with SAD vs. HI) did not significantly affect MAO-A V_T_. Thus, effects of rs1386494 on MAO-A V_T_ appear not to be pathophysiologically relevant. However, based on its pronounced effect (26% higher levels in CC homozygotes) we suggest considering the relevance of this genetic variant as a cofactor when modeling MAO-A V_T_ changes in future studies. Furthermore, the relevance of the relation between rs1386494 and MAO-A V_T_ in other phenomena also associated with altered MAO-A such aggression [44] and suicidality [45], should be assessed.

One limitation of this study is the relatively small sample size, though typical of PET studies with [^11^C]harmine [8, 9]. In addition, the variants selected here are SNPs published in the context of candidate gene studies, which, as discussed previously [46], carries limitations. In addition, we acknowledge that the SNPs assessed here do not represent an exhaustive list of potentially relevant variants. Alternative polygenetic or even genome-wide approaches are not feasible in a study of this size [47]. In addition, criticism of the candidate gene approach may be more applicable to clinical studies, with imaging genetics studies elucidating larger effect sizes than their clinical counterparts [48]. Furthermore, while the clinical relevance of a single variant may be limited, mechanistic investigation of single-variant effects on human in vivo expression, e.g. assessed with PET, does indeed substantiate our understanding of genetic mechanisms influencing brain function. Interestingly, *TPH2* was recently isolated as a top susceptibility gene on suicidality in depression [49]. Furthermore, though assessment of genetic variant effects on MAO-A V_T_ in individuals with SAD is intuitive based on previous studies showing sensitivity of the serotonin system to season [39] and light [50–52], clinical studies associating rs1386494 with disease risk were performed in non-seasonal depression [15]. Thus, though we were able to glean knowledge regarding the neurobiological correlates of rs1386494, the pathophysiologic relevance, or in this case lack there-of, for non-seasonal depression is not definitive.

## Conclusion

We demonstrate that rs1386494, a common *TPH2* variant previously linked to depression [15], suicidality [34, 41], and antidepressant response [53], is associated with substantially altered MAO-A levels in humans in vivo. The effect of *TPH2* genotype on MAO-A V_T_ might occur on the level of their common monoaminergic products/substrates. The effect of monoamine levels on MAO-A activity was previously demonstrated in vivo [11] and in vitro [43]. Alternatively, co-inheritance of another genetic factor with known effects on 5-HT homeostasis [23], may play a mechanistic role. Results were not diagnosis-specific, suggesting that the clinical effects rs1386494 mediates, for example in depression, are not related to MAO-A V_T_. Our results provide insight into how genetic variant effects within serotonin turnover translate to the cerebral serotonin system.

## Supplementary information

Figure S1: Effects of serotonin turnover variants on MAO-A V_T_ (only fall/winter scans)

Table S1: General linear model results (all scans)

Table S2: Genotype grouping and frequencies (only fall/winter scans)

Table S3: Genotype effects on global MAO-A V_T_ (only fall/winter scans)

Table S4: General linear model results (only fall/winter scans)
