# Supplementary material for: Impact of genetic variants within serotonin turnover enzymes on human cerebral monoamine oxidase A in vivo
Source: Transl Psychiatry. 2023 Jun 15;13:208. doi: 10.1038/s41398-023-02506-2 (PMC10272199; doi:10.1038/s41398-023-02506-2)
Supplement: Supplementary file 1 — Figure S1: Effects of serotonin turnover variants on MAO-A VT (only fall/winter scans) [file 41398_2023_2506_MOESM1_ESM.docx]

**Figure S1: Effects of serotonin turnover variants on MAO-A V_T_ (only fall/winter scans)**


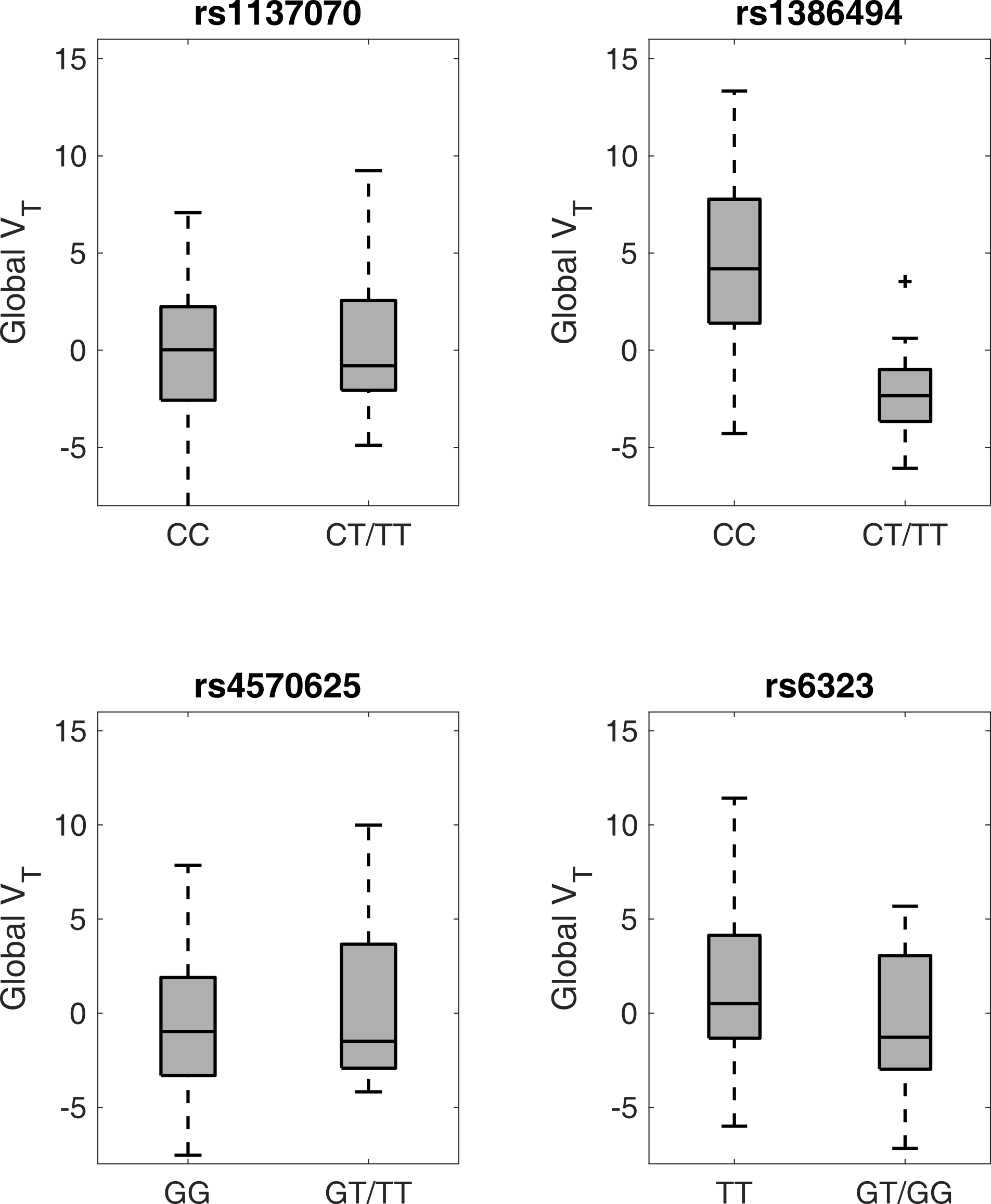


MAO-A V_T_ was significantly (F_1, 124.31_ = 9.49, p_corr_ = 0.02, cohen’s d: 0.90, approx. 28% numerically) higher in rs1386494 (*TPH2*) CC homozygotes than in T carriers. The other assessed genotypes (*MAOA*: rs1137070, rs6323, and *TPH2*: rs4570625) did not demonstrate statistically significant effects on MAO-A V_T_. MAO-A V_T_ represented here is corrected for age, sex, and group (individuals with SAD/HI). Here, rs1137070 and rs2064070 were in perfect LD, thus only rs1137070 is reported. Analyses excluding 4 HI in whom only spring/summer scans were available. Boxes represent 50% of data, median depicted, whiskers represent range, + denotes outlier.
