# Supplementary material for: Impact of genetic variants within serotonin turnover enzymes on human cerebral monoamine oxidase A in vivo
Source: Transl Psychiatry. 2023 Jun 15;13:208. doi: 10.1038/s41398-023-02506-2 (PMC10272199; doi:10.1038/s41398-023-02506-2)
Supplement: Supplementary file 2 — Table S1: General linear model results (all scans) [file 41398_2023_2506_MOESM2_ESM.docx]

**Table S1: General linear model results (all scans)**

|  | rs1386494 | | | rs1137070 | | | rs6323 | | | rs4570625 | | |
| --- | --- | --- | --- | --- | --- | --- | --- | --- | --- | --- | --- | --- |
| Variable | F | Sig. | Sig. corr | F | Sig. | Sig. corr | F | Sig. | Sig. corr | F | Sig. | Sig. corr |
| ZAge | 0.27 | 0.61 | 2.66 | 0.30 | 0.59 | 2.58 | 0.30 | 0.59 | 2.58 | 0.28 | 0.60 | 2.66 |
| SNP | 9.52 | 0.003 | 0.01 | 0.01 | 0.96 | 3.85 | 0.63 | 0.43 | 1.73 | 0.14 | 0.71 | 2.83 |
| Group | 1.09 | 0.30 | 1.21 | 1.20 | 0.28 | 1.12 | 0.91 | 0.35 | 1.38 | 1.30 | 0.26 | 1.04 |
| Sex | 0.87 | 0.36 | 1.42 | 0.51 | 0.48 | 1.91 | 0.96 | 0.33 | 1.33 | 0.48 | 0.49 | 1.97 |

*rs1137070 and rs2064070 were in perfect LD, thus only rs1137070 is reported
