# Supplementary material for: Impact of genetic variants within serotonin turnover enzymes on human cerebral monoamine oxidase A in vivo
Source: Transl Psychiatry. 2023 Jun 15;13:208. doi: 10.1038/s41398-023-02506-2 (PMC10272199; doi:10.1038/s41398-023-02506-2)
Supplement: Supplementary file 3 — Table S2: Genotype grouping and frequencies (only fall/winter scans) [file 41398_2023_2506_MOESM3_ESM.docx]

**Table S2: Genotype grouping and frequencies (only fall/winter scans)**

| **Variant** |  |  |
| --- | --- | --- |
| *MAOA* | **n (m, f)** | **n (m, f)** |
| rs1137070 (T/C)* | CC | CT, TT |
|  | 31 (16, 15) | 16 (3, 13) |
| rs6323 (G/T) | TT | TG, GG |
|  | 32 (16, 16) | 15 (3, 12) |
| *TPH2* | **n** | **n** |
| rs1386494 (T/C) | CC | CT |
|  | 31 | 16 |
| rs4570625 (T/G) | GG | GT, TT |
|  | 26 | 21 |

*rs1137070 and rs2064070 were in perfect LD, thus only rs1137070 is reported

*MAOA* SNPs are split by sex based on X-linkage.
