# Supplementary material for: Impact of genetic variants within serotonin turnover enzymes on human cerebral monoamine oxidase A in vivo
Source: Transl Psychiatry. 2023 Jun 15;13:208. doi: 10.1038/s41398-023-02506-2 (PMC10272199; doi:10.1038/s41398-023-02506-2)
Supplement: Supplementary file 4 — Table S3: Genotype effects on global MAO-A VT (only fall/winter scans) [file 41398_2023_2506_MOESM4_ESM.docx]

**Table S3: Genotype effects on global MAO-A V_T_ (only fall/winter scans)**

| **Variant** | **Genotype** | **Global MAO-A V_T_ (mean +/- SD)** |
| --- | --- | --- |
| *MAOA* |  |  |
| rs1137070 (T/C)* | CC | 15.06 ± 3.72 |
|  | CT, TT | 15.19 ± 4.41 |
| rs6323 (G/T) | TT | 15.42 ± 4.19 |
|  | TG, GG | 14.43 ± 3.31 |
| *TPH2* |  |  |
| rs1386494 (T/C) | CC | 16.31 ± 3.96 |
|  | CT | 12.78 ± 2.65 |
| rs4570625 (T/G) | GG | 14.90 ± 4.05 |
|  | GT, TT | 15.37 ± 3.83 |

*rs1137070 and rs2064070 were in perfect LD, thus only rs1137070 is reported. Values uncorrected for covariates.
