# Supplementary material for: Impact of genetic variants within serotonin turnover enzymes on human cerebral monoamine oxidase A in vivo
Source: Transl Psychiatry. 2023 Jun 15;13:208. doi: 10.1038/s41398-023-02506-2 (PMC10272199; doi:10.1038/s41398-023-02506-2)
Supplement: Supplementary file 5 — Table S4: General linear model results (only fall/winter scans) [file 41398_2023_2506_MOESM5_ESM.docx]

**Table S4: General linear model results (only fall/winter scans)**

|  | rs1386494 | | | rs1137070 | | | rs6323 | | | rs4570625 | | |
| --- | --- | --- | --- | --- | --- | --- | --- | --- | --- | --- | --- | --- |
| Variable | F | Sig. | Sig. corr | F | Sig. | Sig. corr | F | Sig. | Sig. corr | F | Sig. | Sig. corr |
| ZAge | 0.19 | 0.66 | 2.66 | 0.22 | 0.64 | 2.58 | 0.22 | 0.64 | 2.58 | 0.19 | 0.67 | 2.66 |
| SNP | 9.49 | 0.004 | 0.02 | 0.00 | 0.99 | 3.97 | 0.76 | 0.39 | 1.55 | 0.18 | 0.68 | 2.70 |
| Group | 0.69 | 0.41 | 1.65 | 1.09 | 0.30 | 1.21 | 0.76 | 0.39 | 1.56 | 1.23 | 0.27 | 1.09 |
| Sex | 0.81 | 0.37 | 1.49 | 0.39 | 0.54 | 2.15 | 0.81 | 0.37 | 1.49 | 0.35 | 0.56 | 2.24 |

*rs1137070 and rs2064070 were in perfect LD, thus only rs1137070 is reported
